# Online Fitness Content as a behavioral catalyst: extending the Theory of Planned Behavior to unmask time-dependent effects on resistance training engagement

**DOI:** 10.3389/fpsyg.2025.1575832

**Published:** 2025-11-20

**Authors:** Wei Wei, Ronghai Su, Chen Lin, Meng Meng, Rongrong Zheng

**Affiliations:** 1 School of Physical Education, China University of Geosciences, Beijing, China; 2 College of Physical Education and Sports, Beijing Normal University, Beijing, China; 3 School of Physical Education, Education University of Hong Kong, Hong Kong, China; 4 Department of Physical Education, Xiamen University, Xiamen, China

**Keywords:** resistance training, social networking sites, Theory of Planned Behavior, Online Fitness Content, Consumption Time

## Abstract

**Introduction:**

Resistance training plays a vital role in promoting physical and mental health among university students. However, approximately three-quarters of students globally fail to meet recommended guidelines, with a significant intention-behavior gap persisting. Based on an extended Theory of Planned Behavior (TPB) framework, this study aimed to examine the dual-chain mediating effects of Online Fitness Content (OFC) on students’ resistance training behavior through Planning (PL) and Positive Emotion (PE), and to test the suppressing effect of Consumption Time (CT).

**Method:**

A stratified random sampling method was administered to 356 college students (60.39% male, 39.61% female) regularly engaged in resistance training across 17 Chinese universities. Structural Equation Modeling (AMOS 20.0) was employed to analyze the chain mediation pathways, with multi-group comparisons testing the moderating effect of CT.

**Results:**

The core TPB variables (attitude, subjective norm, perceived behavioral control) significantly predicted intention (path coefficients: 0.265–0.494, *p* < 0.01), and intention significantly predicted behavior (*β* = 0.229, *p* = 0.032). OFC demonstrated significant dual-path mediation effects through both PL (*β* = 1.215, *p* = 0.001) and PE (*β* = 1.074, *p* = 0.001). The key finding revealed a suppressing effect when daily usage exceeded 30 min: the path effect from OFC to PL attenuated by 72% (*β* > 1 h = 0.192 vs. *β* < 1 h = 1.145), the path effect from OFC to PE attenuated by 73% (*β* > 1 h = 0.301 vs. *β* < 1 h = 1.101), and the direct effect of OFC on behavior became significantly negative (*β* > 1 h = −0.690, *p* < 0.05).

**Discussion:**

The study demonstrates that OFC serves as an effective digital mediator in bridging the intention-behavior gap in university students’ resistance training, but its efficacy exhibits a critical threshold (30 minutes/day). Future health communication should optimize content distribution strategies and implement CT control mechanisms to achieve sustainable behavior change.

## Introduction

Resistance training plays a vital role in promoting physical and mental health, enhancing muscular strength, bone density, and metabolic function, while reducing the risks of chronic diseases (Westcott, 2012; Cavarretta et al., 2018; Wald et al., 2014). The university period represents a critical window for establishing lifelong health behaviors (Snedden et al., 2019; Mehri et al., 2016). Promoting participation in resistance training among college students not only enhances their physical and mental well-being but also fosters the development of sustainable exercise habits into adulthood. However, despite explicit recommendations from both the WHO and ACSM–AHA guidelines that university students should engage in resistance training at least twice per week (World Health Organization, 2020), approximately three-quarters of students worldwide fail to meet these standards (Ren et al., 2024; Garcia-Hermoso et al., 2023). Notably, participation rates in resistance training remain significantly lower than those in aerobic exercise (Abildso et al., 2023; Bennie et al., 2019, 2021). Moreover, a significant discrepancy between self-reported and objectively measured resistance training engagement among university students highlights a pronounced intention-behavior gap. Specifically, while 70.8% of students claimed to exercise at the gym regularly, objective records indicated that only 29.2% actually participated in resistance training, a disparity of 48.3% (Brenner and DeLamater, 2014). Therefore, bridging this intention-behavior gap has emerged as a critical challenge for improving adherence to resistance training programs in this population.

In recent years, social networking sites have provided a new mediating perspective for research on resistance training behaviors due to their unique advantages in information dissemination, social interaction, and virtual environment construction (Kong et al., 2021; Lau et al., 2022). The penetration rate of social networking sites among university students exceeds 90%, with a daily average usage time ranging from 1 to 2.5 h (Twenge and Martin, 2020; Bottaro et al., 2024). However, information overload and variable content quality on social networking sites often lead users to place greater trust in influential accounts within specific domains—a phenomenon also observed in the context of resistance training (Durau et al., 2022; Lutkenhaus et al., 2019; Wiedmann and von Mettenheim, 2020). Studies indicate that workout plans shared by fitness influencers and athletes on platforms such as TikTok and Instagram attract considerable attention among university students and serve as a significant motivator for their engagement in physical exercise (Picazo-Sánchez et al., 2022; O’Donnell et al., 2023). Moreover, the duration of social networking sites usage exhibits complex relationships with user intentions and behaviors (Hall and Liu, 2022; Qin et al., 2024). This is particularly relevant as online fitness content (OFC) on social networking sites is undergoing a shift in values from “thinness-oriented” to “strength and health-focused” paradigms, creating new opportunities to bridge the intention–behavior gap. OFC broadly refers to online graphic, text, and video content designed to promote health, athletic physiques, and healthy lifestyles through exercise, diet, and other methods (Ganson et al., 2023). Its functions primarily encompass two aspects: first, fitness activity support, which involves recording training intensity, frequency, and adherence to form an individual behavior log, reflecting stable user behavior patterns; and second, social interaction and knowledge sharing, which relies on user-generated content such as text, images, and videos (Wang et al., 2024). Furthermore, “fitspiration,” a common form of OFC, motivates users to achieve health goals through fitness images and videos, inspirational quotes, and sharing of personal experiences, further expanding the diverse expression of OFC in behavior promotion (Wang et al., 2024). Nevertheless, existing research has primarily focused on how OFC influences individuals’ intrinsic motivations, such as through Self-Determination Theory (SDT), examining how OFC fulfills users’ needs for competence, autonomy, and relatedness (Wei et al., 2021; Zhang et al., 2024). While these studies elucidate OFC’s role in driving intrinsic motivation for resistance training, they often overlook the integrated mechanisms through which internal and external factors jointly influence behavior. In contrast, the Theory of Planned Behavior (TPB) provides a more systematic framework for explaining behavioral mechanisms via a comprehensive analysis of attitude (an individual’s positive or negative evaluation of the behavior), subjective norms (perceived social pressure from significant others), perceived behavioral control (the perceived ease or difficulty of performing the behavior), and intention (the individual’s readiness to perform the behavior) (Bosnjak et al., 2020). Among these, intention and attitude emphasize internal drives, whereas subjective norms and perceived behavioral control underscore external influences (Ajzen, 2011). Against this backdrop, the present study employs an extended TPB model incorporating both planning and affective variables to investigate the transformation from intention to behavior in university students’ resistance training participation from dual cognitive-affective perspectives. Specifically, OFC caters to personalized needs (Cavaliere, 2024; Martin, 2023; Sweet et al., 2014), compensates for the lack of professional guidance within academic settings, facilitates training planning, and enhances the likelihood of behavioral translation (Kaplan and Haenlein, 2010; López-Carril et al., 2025). On the other hand, real-time interactive features on social networking sites, such as liking and commenting on OFC may elicit positive emotions in users (Maher et al., 2016). These positive emotions are susceptible to social interactions (Zielinski et al., 2023; Rothman and Magee, 2016), act as a powerful force in shaping decision-making and motivation, thereby significantly promoting exercise behaviors (Cameron et al., 2015). The cognitive and emotional changes driven by OFC may shorten the decision-making cycle from intention to behavior. This can address the limitations of the TPB in explaining the low translation rate of intentions into resistance training behaviors (Bosnjak et al., 2020; Armitage, 2005; Ajzen, 2011; Gholamnia-Shirvani et al., 2018; Sheeran and Webb, 2016; Conner and Norman, 2022; Rhodes and Dickau, 2012).

Based on the above, this study constructs an extended TPB model to examine the chained mediation effects between intention and behavior in university students’ resistance training participation, and proposes the following primary hypotheses:

*H1:* Online Fitness Content-driven training plan formulation plays a chained mediating role between resistance training intentions and behaviors among university students.

*H2:* Online Fitness Content-driven positive emotions play a chained mediating role between resistance training intentions and behaviors among university students.

Although OFC has the potential to promote behavioral transformation in resistance training, inter-individual differences in the duration of social media use may significantly influence this effect. Studies have shown that the duration of social media use is negatively correlated with exercise behavior (Hall and Liu, 2022; Hall et al., 2018). Exceeding 2 h of daily social media use can reduce vigorous-intensity physical activity (MVPA) by 5–10 min per day, infringe on daily activity time, increase sedentary time (average 19.8 min), and even shorten sleep duration, further impairing next-day exercise participation (Yao Lin and Lachman, 2022; Herman, 2024; Kontostoli et al., 2023). Furthermore, prolonged eye use and prolonged sitting can strain vision and the lumbar spine (Bener et al., 2013; Kawashima et al., 2016). Other studies have found that limiting social media usage to 30–50 min significantly promotes the conversion of online information into actual behavior (Lutkenhaus et al., 2019; Liu et al., 2024; Bai et al., 2023). However, existing research has primarily focused on the impact of general health information, and direct evidence linking exposure to specific content, such as the OFC, and behavioral conversion remains insufficient (van Duin et al., 2025). Although research has demonstrated that viewing fitness content can enhance motivation, the conversion of motivation into behavior is mediated by multiple factors, with viewing time as a key variable remaining underexplored, limiting our understanding of the mechanisms that influence behavior (Lavoie et al., 2025; Sokolova and Perez, 2021). Therefore, further research is urgently needed to examine the differential effects of varying OFC exposure on intention and behavior.

In summary, this study further proposes the secondary hypothesis:

*H3:* Online Fitness Content Consumption Time moderates the relationship between Online Fitness Content-driven resistance training intentions and behaviors among university students. Specifically, the pathways INT → OFC → PL → BE and INT → OFC → PE → BE follow the same directional trend, but both differ from the INT → OFC → BE pathway. Furthermore, Social Networking Site Consumption Time moderates the relationships between OFC and PL/PE/BE across the three pathways.

**Figure 1:**
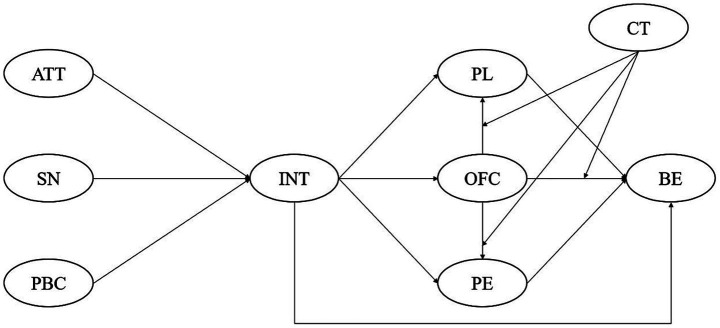
Extended Theory of Planned Behavior model. H1: INT → OFC → PL → BE; H2: INT → OFC → PE → BE; H3: The pathways INT → OFC → PL → BE and INT → OFC → PE → BE follow the same directional trend, but differ from the INT → OFC → BE pathway. Moreover, CT moderates the relationships between OFC and PL/PE/BE across the three pathways. ATT, Attitude; SN, Subjective Norm; PBC, Perceived Behavioral Control; INT, Intention; BE, Behavior; OFC, Online Fitness Content; PL, Plan; PE, Positive Emotion; CT, Consumption Time.

The proposed extended Theory of Planned Behavior model is illustrated in Figure 1.

## Methods

### Participants and research design

Using R (version 4.2.2), based on the recommended calculation method for Structural Equation Modeling (SEM) based on the RMSEA approach (MacCallum et al., 1996), we computed the required sample size as follows: to ensure close fit (*ε*₀ = 0.05 and εₐ = 0.08), the minimum sample size was 54 participants; to test not-close fit (ε₀ = 0.05 and εₐ = 0.01), the minimum sample size was at least 80 participants. As the model included multiple observed indicators, the degrees of freedom (df = 420) were determined by the difference between the number of model parameters and the imposed constraints. Allowing for a 20% rate of invalid responses, we planned to recruit more than 300 participants to ensure adequate statistical power.

A stratified random sampling design was employed. Participants were selected from 17 universities (8 in northern China and 9 in southern China) using stratified random sampling, based on the list of standard universities published by the Ministry of Education of China. Although dissemination used convenient channels, the randomness of the sample was mainly ensured by the initial stratified sampling of universities. Recruitment notices containing QR codes and detailed informed consent information were distributed via WeChat groups by fitness club leaders at each participating institution. Participants were required to read and agree to the study overview, privacy protection measures, and voluntary participation statement before accessing the questionnaire. All data were collected anonymously and used solely for the purposes of this study. The research team strictly adhered to data confidentiality and storage protocols to safeguard participant privacy. Scanning the QR code directed participants to the questionnaire, whose landing page presented the study overview, privacy measures, and voluntary participation statement. Data collection took place between March and May 2023, with the questionnaire requiring approximately 15 min to complete.

Eligibility Criteria were: good health (no major diseases or current medication use); non-sports-related major; and being a university student who regularly engaged in resistance training (≥2 days/week for at least 3 months) (World Health Organization, 2010). A total of 416 questionnaires were initially collected (297 from northern China and 119 from southern China), achieving a 100% response rate. After manually reviewing the responses, 60 invalid responses were excluded, resulting in 356 valid samples. The average age of participants was 21.37 ± 2.40 years (males: 21.53 ± 2.40 years, n = 215; females: 21.12 ± 2.26 years, n = 141). The top three Social Networking Sites used by participants were TikTok (56.18%), Bilibili (55.62%), and WeChat (46.63%). Additionally, 38.48% of participants reported browsing fitness-related content on Social Networking Sites for ≤0.5 h per day (see Table 1 for details).

**Table 1:** Descriptive analysis of the sample.

Variable	Category	Number	Percentage
Gender	Male	215	60.39%
	Female	141	39.61%
Judgment	Yes	356	100%
CT	≤0.5 h	137	38.48%
	0.5 h–≤1 h	95	26.69%
	1 h–≤2 h	70	19.66%
	2 h–≤3 h	26	7.30%
	>3 h	28	7.87%
OFC_a_	WeChat	166	46.63%
	Little Red Book	161	45.22%
	Microblog	86	24.16%
	Tik Tok	200	56.18%
	Bilibili	198	55.62%
	Tencent	33	9.27%
	Kuaishou	28	7.87%
	Others	22	6.18%
Major	Engineering	125	35.11
	Management	142	39.89
	Arts and Humanities	36	10.11
	Law	36	10.11
	Education	11	3.09
	Natural Sciences	6	1.69

Judgment = “Do I usually use online media software to learn about resistance training-related content?”; CT = “How much time do I usually spend each day browsing resistance training-related videos, pictures, etc., on OFC?”; a represents multiple-choice questions.

### Measures

#### The Theory of Planned Behavior

The Theory of Planned Behavior (TPB) scale was adapted from Ajzen’s standard questionnaire (Ajzen, 2005), utilizing standardized wording from previous studies. The adaptations aimed to enhance the situational relevance for the target behavior. For instance, the original attitude item “I believe engaging in the behavior is/is not valuable” was modified to “I believe spending some time each week on resistance training will be/will not be worthwhile” (3 items), thereby specifying the resistance training context. Subjective norms were assessed using items such as “Most of my family members approve of me spending some time each week on resistance training over the next three months” (4 items), which emphasized the influence of significant others related to resistance training. Perceived behavioral control included items like “I am confident that I can spend some time each week on resistance training over the next three months” (6 items), enhancing the focus on perceived executability. Intention: Included three items, such as “I intend to spend some time each week engaging in resistance training over the next three months.” All TPB items were rated on a 7-point Likert scale (1 = strongly disagree, 7 = strongly agree). Behavior was assessed using two items: “On average, how often did you engage in strength training in the past?” and “How many times have you engaged in strength training in the past two weeks?.” The reliability of the scale was tested using McDonald’s Omega (*ω*). The results indicated values of 0.877 for attitude, 0.973 for subjective norms, 0.953 for perceived behavioral control, and 0.966 for intention. These values meet the reliability standards suggested by Peters (2014), demonstrating that the TPB scale has good reliability.

#### Online Fitness Content

The Online Fitness Content Scale was developed with reference to the Social Media Marketing Activities (SMMA) Scale and the Social Networking Sites Usage & Needs (SNSUN) Scale (Ruangkanjanases et al., 2022; Ali et al., 2019). The scale consists of two dimensions: Online Fitness Content Influence and Online Fitness Content Motivation. Online Fitness Content Influence: Includes three items, such as “I began following more fitness/sports influencers on social media.”; Online Fitness Content Motivation: Includes three items, such as “Watching fitness-related content on social media frequently makes me feel more motivated to train.” The scale uses a 7-point Likert scoring system (1 = strongly disagree, 7 = strongly agree). The reliability of the scale was tested using McDonald’s Omega (*ω*). The results showed a value of 0.835 for Social Networking Sites Influence and 0.973 for Social Networking Sites Motivation, meeting the reliability criteria outlined by Peters (2014). These findings indicate that the Online Fitness Content scale has excellent reliability.

#### Planning

The Planning Scale was adapted from the studies by Sniehotta et al. (2006) and Sniehotta et al. (2005), comprising two dimensions: Action Planning and Coping Planning. Action Planning: Includes five items, such as “I am confident that I have planned the start time for my resistance training program.” Coping Planning: Includes five items, such as “I have considered potential situations in the future, such as academic obligations, that might interfere with completing my training plan.” All items were rated on a 7-point Likert scale (1 = strongly disagree, 7 = strongly agree). The reliability of the scale was tested using McDonald’s Omega (*ω*). The results showed a value of 0.933 for Action Planning and 0.948 for Coping Planning, meeting the reliability standards outlined by Peters (2014). These findings indicate that the Planning Scale has excellent reliability.

#### Positive emotion scale

The assessment of emotions was developed based on The Profile of Mood States (POMS; Searight and Montone, 2017) and the measurement approach by Changiz Mohiyeddini et al. (Jones et al., 2005), using high-frequency emotion words to capture affective experiences associated with the intention to perform resistance training. A 7-point frequency scale (1 = never, 7 = always) was developed and administered to a healthy population with prior resistance training experience (*n* = 169; 67 males, 102 females). From the 40 emotion words across the six POMS dimensions: (Westcott, 2012) tension/anxiety, (Cavarretta et al., 2018) anger/hostility, (Wald et al., 2014) vigor/activity, (Snedden et al., 2019) fatigue/inertia, (Mehri et al., 2016) depression/dejection, and (World Health Organization, 2020) confusion—the top 10 most frequently reported terms related to resistance training intention were selected: Cheerful, Lively, Active, Enthusiastic, Energetic, Competent, Confident, Satisfied, Full of Pep, and Vigorous. All selected terms reflected positive emotions. Exploratory factor analysis indicated strong correlations among the 10 emotion indicators, which loaded onto a single common factor, accounting for a cumulative variance of 65.15%. The scale’s content validity was evaluated by a panel of six experts in strength conditioning and sport psychology, who assessed the relevance, appropriateness, validity, and feasibility of the selected emotion terms, yielding an average score of 87.18. Reliability analysis demonstrated excellent internal consistency, with McDonald’s Omega (*ω*) = 0.974, indicating that the positive emotion scale possesses strong psychometric properties.

### Data analyzes

To reduce model complexity and enhance measurement precision, item parceling was performed separately for the constructs of the Online Fitness Content scale and the planning scale, using the content grouping approach. This involved grouping items according to the theoretical dimensions of the scales (e.g., the influence and motivation dimensions of the Online Fitness Content scale) and calculating the mean score of each group to serve as a parcel indicator. The validated measurement model and structural model were analyzed using structural equation modeling with AMOS 20.0 software. Model parameters were estimated using the maximum likelihood (ML) method, which assumes a multivariate normal distribution of the data and provides robust parameter estimates. As the data were primarily collected via self-report measures, which are susceptible to social desirability effects, testing for common method bias (CMB) was necessary. First, Harman’s single-factor test was conducted. The results indicated poor model fit when all items were loaded onto a single common factor: *χ*^2^/df = 12.023, RMSEA = 0.176, CFI = 0.691, GFI = 0.348, TLI = 0.423, SRMR = 0.090, suggesting that CMB was not a severe concern. Considering the limited sensitivity of Harman’s test, the unmeasured latent method factor approach was further employed. This method involved introducing a method factor uncorrelated with the substantive latent variables into the original seven-factor model and allowing all items to load on this factor. The model including the method factor demonstrated good fit: *χ*^2^/df = 2.483, RMSEA = 0.07, CFI = 0.960, GFI = 0.860, TLI = 0.870, SRMR = 0.045. However, the chi-square difference test was not statistically significant (*p* > 0.05), indicating that the model incorporating the method factor did not yield a significantly better fit than the original seven-factor model. This further confirmed that common method bias was not a substantial issue in this study. The significance level for all hypothesis tests was set at *α* < 0.05.

## Results

### Confirmatory factor analyses

A confirmatory factor analysis (CFA) was conducted for the seven variables: OFC, attitude, subjective norms, perceived behavioral control, intention, planning, and positive emotion. The Corrected Item-Total Correlation (CITC) for all measurement items or indicators exceeded 0.30, indicating good reliability of the scales (Zhang et al., 2020; Churchill and Peter, 1984). Factor loadings ranged from 0.620 to 0.968, all above the 0.50 threshold. Composite reliability (CR) values ranged from 0.814 to 0.973, exceeding the 0.60 benchmark, while the average variance extracted (AVE) values ranged from 0.690 to 0.901, all above the 0.50 criterion (Hair, 1998); see Table 2. The seven-factor model demonstrated good model fit: *χ*^2^/df = 2.369, RMSEA = 0.06, CFI = 0.970, GFI = 0.920, TLI = 0.920, SRMR = 0.038. Therefore, the measurement model exhibited satisfactory reliability and convergent validity.

**Table 2:** Confirmatory factor analysis of measures.

Variables	Items	CITC	Std.	SMC	CR	AVE
ATT	ATT1	0.539	0.907	0.823	0.884	0.72
	ATT2	0.489	0.931	0.867		
	ATT3	0.553	0.687	0.472		
SN	SN1	0.531	0.909	0.826	0.973	0.901
	SN2	0.494	0.956	0.914		
	SN3	0.593	0.968	0.937		
	SN4	0.476	0.962	0.925		
PBC	PBC1	0.602	0.937	0.878	0.952	0.769
	PBC2	0.562	0.935	0.874		
	PBC3	0.702	0.934	0.872		
	PBC4	0.567	0.8	0.64		
	PBC5	0.537	0.838	0.702		
	PBC6	0.632	0.806	0.65		
INT	INT1	0.519	0.892	0.796	0.952	0.868
	INT2	0.533	0.956	0.914		
	INT3	0.507	0.946	0.895		
OFC	Influence	0.604	0.719	0.517	0.814	0.69
	Impulse	0.592	0.929	0.863		
PL	Action	0.584	0.875	0.766	0.894	0.809
	Cope	0.627	0.923	0.852		
PE	Cheerful	0.577	0.872	0.76	0.968	0.752
	Lively	0.622	0.873	0.762		
	Active	0.557	0.885	0.783		
	Enthusiastic	0.509	0.891	0.794		
	Energetic	0.585	0.873	0.762		
	Competent	0.562	0.879	0.773		
	Confident	0.547	0.857	0.734		
	Satisfied	0.652	0.911	0.83		
	Pep	0.547	0.875	0.766		
	Vigorous	0.552	0.745	0.555		

CITC, Corrected Item Total Correlation; Std., Standardized; SMC, Squared Multiple Correlation; CR, Composite Reliability; AVE, Average Variance Extracted; ATT, Attitude; SN, Subjective Norm; PBC, Perceived Behavioral Control; INT, Intention; BE, Behavior; OFC, Online Fitness Content; PL, Plan; PE, Positive Emotion; The letters 1–6 after the abbreviation represent different items.

### Analysis of discriminative validity

The seven variables—OFC, attitude, subjective norms, perceived behavioral control, intention, planning, and positive emotion—all exhibited significant positive correlations (*γ* = 0.634–0.845, *p* < 0.001). These correlation statistics provided preliminary support for subsequent hypothesis testing. Discriminant validity was assessed using the AVE method (Fornell and Larcker, 1981), which requires that the square root of the AVE for each variable exceed the correlation coefficients between all paired variables. As shown in Table 3, the square roots of the AVEs (ranging from 0.831 to 0.949, displayed on the diagonal) were greater than all corresponding inter-variable correlation coefficients, supporting discriminant validity among the constructs. These results indicate that the variables included in this study possess adequate discriminant validity and represent distinct constructs.

**Table 3:** Pearson correlation and discriminant validity.

	1	2	3	4	5	6	7
1. OFC	0.831						
2. ATT	0.643***	0.849					
3. SN	0.675***	0.765***	0.949				
4. PBC	0.702***	0.778***	0.822***	0.877			
5. INT	0.732***	0.776***	0.804***	0.845***	0.932		
6. Plan	0.735***	0.690***	0.720***	0.774***	0.769***	0.899	
7. Emotion	0.678***	0.676***	0.634***	0.668***	0.703***	0.723***	0.867

The diagonal bold values represent the square root of the AVE, and the lower triangle contains the Pearson correlations between variables. *** indicates *p* < 0.001; ATT, Attitude; SN, Subjective Norm; PBC, Perceived Behavioral Control; INT, Intention; BE, Behavior; OFC, Online Fitness Content; PL, Plan; PE, Positive Emotion.

### Model fit and path analysis of the Theory of Planned Behavior

**Figure 2:**
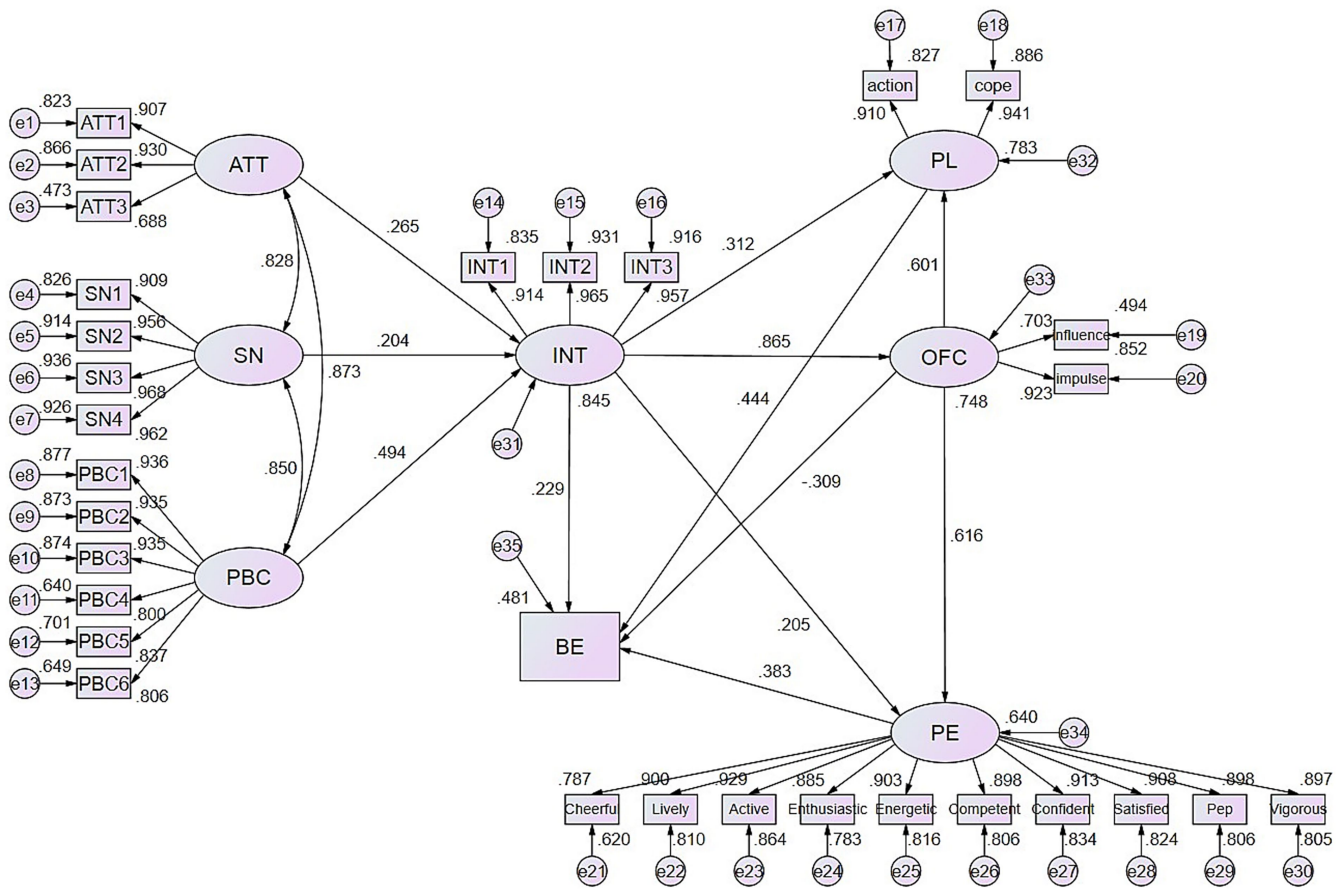
Structural Equation Model analysis diagram. ATT, Attitude; SN, Subjective Norm; PBC, Perceived Behavioral Control; INT, Intention; BE, Behavior; OFC, Online Fitness Content; PL, Plan; PE, Positive Emotion.

To address potential model fit issues arising from large sample sizes (*N* > 200) in Structural Equation Modeling (SEM), which can inflate the chi-square statistic, bootstrap correction was applied to the model fit indices. The bootstrap-corrected model demonstrated adequate fit: *χ*^2^ = 715.83, *χ*^2^/df = 1.70, CFI = 0.98, GFI = 0.95, NNFI = 0.98, IFI = 0.98, TLI = 0.98, RMSEA = 0.04. These results indicate satisfactory model fit, as illustrated in Figure 2. Path analysis results are presented in Table 3. Attitude significantly predicted intention (*β* = 0.265, *Z* = 4.156, 95% CI [0.068, 0.404], *p* < 0.001), as did subjective norms (*β* = 0.204, *Z* = 3.874, 95% CI [0.066, 0.343], *p* = 0.002) and perceived behavioral control (*β* = 0.494, *Z* = 7.557, 95% CI [0.282, 0.718], *p* < 0.001). Furthermore, intention significantly predicted behavior (*β* = 0.229, *Z* = 2.319, 95% CI [0.122, 3.175], *p* < 0.05). These findings collectively lend support to the appropriateness of employing the Theory of Planned Behavior as the theoretical foundation for this study.

### Testing hypotheses of the TPB

The bootstrapping method, which offers greater statistical power than the causal steps approach or product of coefficients methods (Williams and MacKinnon, 2008), was employed to test the mediation effects in the model, using 5,000 bootstrap samples (see Table 4). The total effect between intention and behavior was significant (*B* = 3.231, *p* < 0.001), as were the direct effect (*B* = 1.205, *p* = 0.032) and the indirect effect (*B* = 2.026, *p* = 0.048). Specifically, the mediating roles of planning (PL: *B* = 0.73, *p* = 0.145) and positive emotion (PE: *B* = 0.414, *p* = 0.305) between intention and behavior were not significant. However, the mediating roles of OFC via planning (*B* = 1.215, *p* = 0.001) and OFC via positive emotion (*B* = 1.074, *p* = 0.001) between intention and behavior were significant, supporting Hypotheses 1 and 2. Notably, the indirect effect of OFC between intention and behavior was negative (*B* = −1.407, *p* = 0.039) and opposite in direction to the direct effect, indicating a suppression effect (Wen and Ye, 2014), warranting further analysis. Daily social media use of 30 min or less typically reflects purposeful, controlled instrumental use, whereas use exceeding 30 min—particularly reaching several hours—often indicates more passive, aimless content consumption and “scrolling” behavior (Verduyn et al., 2017; Frison and Eggermont, 2020). This usage pattern aligns more closely with problematic social media use, potentially substituting for real-world social interaction and reflecting poor time management (Marino et al., 2018; Boer et al., 2022). Therefore, participants were dichotomized into two groups for multi-group SEM analysis: those spending ≤30 min per day on social networking sites (coded as 1) and those spending >30 min (coded as 2). Results are presented in Table 5. A significant negative indirect effect of OFC was found between intention and behavior. Using the ≤30 min group as the reference, the >30 min group showed lower predictive effects of OFC on planning (*B* = 0.399, SE = 0.089) and positive emotion (*B* = 0.439, SE = 0.101), ultimately resulting in a negative predictive effect on resistance training behavior (*B* = −0.690, SE = 0.672). Significant differences between the two groups were observed in the paths from OFC to planning (Δ*χ*^2^ = 5.775, *p* = 0.016), OFC to positive emotion (Δ*χ*^2^ = 5.141, *p* = 0.023), and OFC to behavior (Δ*χ*^2^ = 4.893, *p* = 0.027). No significant difference was found in the path from intention to OFC (Δ*χ*^2^ = 0.061, *p* = 0.806). In summary, the weaker effects of OFC on planning and positive emotion, coupled with its negative effect on behavior in the >30 min group, counteracted the positive effects observed in the ≤30 min group. This resulted in an overall negative indirect effect of OFC, indicating that the pattern in the high-usage group suppressed the relationship between intention and behavior. Consequently, Hypothesis 3 was supported.

**Table 4:** Mediating effect test between intention and behavior.

Level 2	Level 1	Path	*B*	*σ*	*Z*	95% CI	*p*	Level 1 result	Level 2 result
		INT to PL to BE	0.73	0.868	0.841	[−0.661, 1.721]	0.145	Not significant	
		INT to PE to BE	0.414	0.523	0.792	[−0.72, 1.006]	0.305	Not significant	
H3		INT to OFC to BE	−1.407	1.894	−0.743	[−5.546, −0.069]	0.039	Significant	Suppression effect
	H1	INT to OFC to PL to BE	1.215	1.49	0.815	[0.382, 4.273]	0.001	Supported	
	H2	INT to OFC to PE to BE	1.074	0.732	1.467	[0.454, 3.001]	0.001	Supported	
Direct effect			1.205	1.133	1.064	[0.122, 3.175]	0.032	Significant	
Total indirect effect			2.026	1.138	1.780	[0.039, 3.206]	0.048	Significant	
Total effect			3.231	0.293	11.027	[2.713, 3.884]	<0.001	Significant	

*B* = Coefficient; *σ* = Standard Error; *Z* = *Z*-score; Bootstrap 5000次; ATT, Attitude; SN, Subjective Norm; PBC, Perceived Behavioral Control; INT, Intention; BE, Behavior; OFC, Online Fitness Content; PL, Plan; PE, Positive Emotion.

**Table 5:** Multi-group comparison of structural equation models for Consumption Time (less than 30 min vs. more than 30 min).

Path	Group	Default model			Model comparisons	
		*B*	*σ*	*Z*	∆*x*^2^(*df*)	*p*
INT to OFC	<30 min	0.771	0.046	16.871	0.061 (1)	0.806 (>0.05)
	>30 min	0.755	0.047	16.200		
OFC to PL	<30 min	0.961	0.232	4.137	5.775 (1)	0.016 (<0.05)
	>30 min	0.399	0.089	4.501		
OFC to PE	<30 min	1.064	0.273	3.897	5.141 (1)	0.023 (<0.05)
	>30 min	0.439	0.101	4.367		
OFC to BE	<30 min	0.935	0.389	−2.404	4.893 (1)	0.027 (<0.05)
	>30 min	−0.690	0.672	−1.026		

*B* = Coefficient; *σ* = Standard Error; *Z* = *Z*-score; ATT, Attitude; SN, Subjective Norm; PBC, Perceived Behavioral Control; INT, Intention; BE, Behavior; OFC, Online Fitness Content; PL, Plan; PE, Positive Emotion.

### Cross-validation of the model

To enhance the robustness of the research findings and test the model’s stability, the total sample was randomly split into two groups, each containing 178 participants. A multi-group structural equation modeling analysis was conducted to examine the cross-validation of the theoretical model by testing for significant differences between these two randomly split samples. Following a sequence from least to most restrictive, five nested models were tested: the measurement weights model, structural weights model, structural covariances model, structural residuals model, and measurement residuals model. The differences in model fit between successive models were analyzed sequentially (Blozis and Craft, 2024). As shown in Table 6, constraining all 27 factor loadings to be equal across groups (measurement weights model) resulted in a *χ*^2^ increase of 38.242 (*p* = 0.074) and a ΔCFI of −0.001, which is below the 0.01 threshold, indicating the factor loadings were invariant. Imposing additional constraints on the 8 structural path coefficients (structural weights model) led to a *χ*^2^ increase of 13.185 (*p* = 0.106) and a ΔCFI of 0.000, supporting the invariance of the structural paths. Further constraining the 6 structural covariances (structural covariances model) yielded a *χ*^2^ increase of 7.649 (*p* = 0.265) and a ΔCFI of −0.001, indicating covariance invariance. Subsequently, constraining the 4 structural residuals (structural residuals model) resulted in a significant *χ*^2^ increase of 22.760 (*p* < 0.001) but a minimal ΔCFI of −0.001, suggesting residual invariance. Finally, constraining the 31 measurement residuals (measurement residuals model) led to a substantial *χ*^2^ increase of 170.614 (*p* < 0.001) and a ΔCFI of −0.009, which remains below the critical threshold. Additionally, the changes in other indices (ΔNFI, ΔIFI, ΔRFI, ΔTLI) were all within acceptable limits (< 0.05), meeting the standards established by Little (1997) and Cheung and Rensvold (2002). These results collectively demonstrate that the theoretical model did not differ significantly between the two randomly split samples, confirming its stability. Furthermore, as indicated in Table 7, the unconstrained model demonstrated superior fit compared to all sequentially constrained models, further affirming the high stability of the model and, consequently, the reliability of the study’s conclusions.

**Table 6:** Cross-validation test of the model.

Model	Δ*df*	Δ*χ^2^*	*p*	ΔNFI	ΔIFI	ΔRFI	ΔTLI	ΔCFI
Measurement weights	27	38.242	0.074	0.002	0.002	−0.002	−0.002	−0.001
Structural weights	8	13.185	0.106	0.001	0.001	0.000	0.000	0.000
Structural covariances	6	7.649	0.265	0.000	0.001	0.000	0.000	−0.001
Structural residuals	4	22.760	0.000	0.001	0.001	0.001	0.001	−0.001
Measurement residuals	31	170.614	0.000	0.011	0.011	0.006	0.007	−0.009

**Table 7:** Multi-group model fit indices.

Model	NPAR	*χ*^2^/*df*	GFI	AGFI	CFI	NFI	IFI	TLI	RMSEA
Unconstrained	152	2.329	0.745	0.699	0.927	0.879	0.927	0.919	0.061
Measurement weights	125	2.301	0.74	0.703	0.926	0.877	0.926	0.921	0.061
Structural weights	117	2.295	0.739	0.704	0.926	0.876	0.926	0.921	0.06
Structural covariances	111	2.288	0.738	0.705	0.925	0.875	0.926	0.921	0.06
Structural residuals	107	2.304	0.736	0.704	0.924	0.874	0.924	0.92	0.061
Measurement residuals	76	2.412	0.719	0.696	0.915	0.863	0.915	0.914	0.063

NPAR, number of parameters.

## Discussion

This research, based on an expanded TPB model, illuminates the dual-faceted role of OFC in influencing the intention-behavior relationship in university students’ resistance training. OFC facilitates the translation of intention into behavior by promoting the formulation and implementation of training plans. It also shortens the decision-making cycle between intention and behavior by fostering positive emotions. However, the suppression effect of OFC becomes evident when usage exceeds 30 min per day, weakening the positive effects of planning and emotions and significantly negatively impacting behavior.

### The dual role of Online Fitness Content within the TPB framework: theoretical extension and critical threshold effect

The primary theoretical significance of this study lies in revealing how OFC integrates into and extends the TPB framework within the social networking sites environment. Unlike prior research highlighting the gap between online participation and offline behavior—exemplified by the Ice Bucket Challenge, where many participants did not mention ALS or donate (Van der Linden, 2017)—this study focused on university students with prior experience in resistance training. The findings indicate that for this population, OFC significantly facilitates the alignment and implementation of intentions into offline behaviors through the mediating pathways of planning and positive emotion, thereby enhancing the consistency between intentions and behaviors. However, this facilitative effect is highly dependent on usage duration. The study further identified a critical threshold: when daily OFC use exceeds 30 min, the positive influence of OFC reverses, exhibiting a significant suppressing effect. This outcome can be interpreted through the lens of information cognitive load and processing. Time serves as a crucial dimension (Oberiri Destiny et al., 2022); while diverse online guidance can encourage active information-seeking and processing strategies, excessive recommendations and choices often lead to cognitive overload (Zhong et al., 2024). Prolonged exposure may exceed an individual’s information processing capacity, resulting in difficulties in cognitive synthesis, increased anxiety, and an impaired ability to filter and absorb high-quality training information. Such informational redundancy not only undermines the efficiency of plan formation but can also obstruct behavioral execution, shifting user focus toward passive content consumption (Cao et al., 2021). Moreover, although interactive features such as likes and comments can elicit positive emotions, excessive use fosters a “reward cycle” that leads to emotional habituation or fatigue, ultimately weakening the motivational drive for behavior execution (Goldenberg and Gross, 2020; Przybylski and Weinstein, 2017). Studies also indicate that frequent online browsing correlates significantly with attentional dispersion (Marty-Dugas et al., 2018), and prolonged overuse is associated with reduced sleep quality and increased psychological burden (Przybylski and Weinstein, 2017; Exelmans and Van den Bulck, 2017), further impairing the foundational conditions for translating intention into action. Thus, the present results corroborate the view that excessive OFC consumption distorts behavioral focus and inhibits the conversion of training intentions into actual behavior.

### Mechanism of the planning pathway

Although the present study found that planning did not significantly mediate the relationship between intention and behavior among university students—a result that contrasts with research emphasizing individual proactive planning (Lidong et al., 2025) or the role of positive mental representations during plan formation as key drivers of intention translation (Lachman and Burack, 2016; Scholz et al., 2008)—it reveals that OFC reshapes the mechanism of behavior transformation by comprehensively intervening in the intention-behavior pathway within the social networking sites environment. By providing content highly aligned with individual preferences of university students (e.g., personalized training programs, demonstrations by fitness influencers), OFC significantly reduces the cognitive cost of plan formulation, thereby serving as a “planning guide” (Gao et al., 2021). This finding is corroborated by related studies indicating that online guidance and monitoring functions—such as video demonstrations, instructional content, goal setting, progress tracking, and behavior evaluation and adjustment (Wang et al., 2020; Geng et al., 2023)—constitute some of the most prevalent and user-preferred features of OFC (Middelweerd et al., 2015; Domin et al., 2022). These features substantially diminish the time, cognitive effort, and cost associated with independent plan development among students (Durau et al., 2022). Furthermore, primary creators of OFC, such as fitness bloggers and online influencers, establish professional knowledge frameworks and instructional content that foster user recognition and brand credibility, thereby facilitating a sustainable cycle of information interaction among users. Consequently, planning behavior derives not only from individual initiative but can also be effectively guided and supplemented through technological means. OFC can thus be regarded as a key mediating variable and driver in the chain linking planning to behavior after the formation of resistance training intentions among university students.

### Mechanism of the emotion pathway: complexity and emotion-dependency

This study also found that the positive emotions elicited by resistance training intentions did not exhibit a significant mediating effect between intention and behavior. This finding contrasts with previous studies suggesting that resistance training improves emotional states (Cavarretta et al., 2018; Cavarretta et al., 2019) or that emotions promote behavior (Mohiyeddini et al., 2009; Weyland et al., 2020). This discrepancy may be attributed to the widespread use of smartphones, wearable devices, the exponential growth of OFC, and the unique characteristics of digital emotional experiences (Penglee et al., 2019; Pope et al., 2019; Raggatt et al., 2018). Some studies suggest that online interaction environments and algorithmic recommendations can amplify users’ emotions, foster emotional convergence and significantly influencing their perception of behavioral value. Additionally, the intensity of emotional expression can predict the level of likes and shares received, further enhancing the impact of positive emotions. For instance, community-driven enthusiasm and resonance triggered by success stories can magnify emotional effects (Goldenberg and Gross, 2020; Gao et al., 2021; Gao et al., 2018; Klier et al., 2022; Goldenberg and Gross, 2020). In this study, the positive emotions induced were linked to university students’ real-life resistance training behaviors. The findings showed that OFC, through precise content recommendations and influencer-driven support, triggered users’ sense of identification and activated emotional states such as cheerfulness, liveliness, activity, and enthusiasm. These emotions played a role in situational activation within decision-making processes. Moreover, the interactive and feedback mechanisms of social networking sites, such as sharing training results, likes, and comments, were found to activate the brain’s reward circuits, thereby reinforcing emotional incentives and strengthening the connection between intentions and behaviors (Sherman et al., 2018). This finding offers a new theoretical perspective for extending the TPB in the context of digital health, suggesting that emotional experiences are not merely outcomes of behavior but also catalysts for behavioral transformation.

### Limitations and future directions

As an exploratory study, this research has several limitations. First, the sample representation is limited, as it only included Chinese university students with prior resistance training experience. Therefore, caution is needed when generalizing the findings to other populations or cross-cultural contexts. Second, although validated measurement tools were used, the assessment of OFC focused primarily on usage duration, without thoroughly examining the potential influence of interactive features or content heterogeneity, such as the quality and frequency of user-content and user-influencer interactions, as well as content variations in information quality, emotional tone, and influencer type. These dimensions may be key contextual factors influencing the translation of behavior. Future research could conduct more precise measurements of the relationship between the volume of OFC information and university students’ information reception and processing capacity to determine specific threshold standards. Third, the cross-sectional survey design limits the ability to establish causal or temporal relationships between variables. Future studies should address these limitations by Westcott (2012) adopting multi-method approaches, such as incorporating objective app usage data to measure key variables; (Cavarretta et al., 2018) deepening the measurement of OFC quality by deconstructing its interactive dimensions and content characteristics; Wald et al. (2014) validating the model in larger and more diverse samples, including theory-based subgroup comparisons; and Snedden et al. (2019) employing longitudinal designs or experimental interventions to verify the effects and critical thresholds of OFC from a causal inference perspective.

### Implications

The study found that OFC significantly facilitates the translation of resistance training intentions into behavior by reducing the cognitive cost of planning. To further enhance its practical value, it is recommended to embed diversified and personalized “planning guide functions” within OFC platforms. Based on individual user characteristics such as gender, fitness level, and training goals, a tiered content system can be dynamically generated. Utilizing check-in systems, progress tracking tools, and achievement rewards can transform plan execution into visible behavioral completion rates, thereby increasing user participation and adherence. Simultaneously, strengthening the professional knowledge output of content creators and influencers is crucial to ensure the scientific reliability of the provided plans, avoiding limitations in user plan effectiveness due to low-quality content.

This study revealed the indirect influence of the emotional pathway on behavioral translation, indicating that OFC can induce and enhance positive emotions to promote the alignment of intention and behavior among university students. It is therefore suggested to design structured emotional incentive feedback mechanisms. Features such as “likes” and “shares” can be leveraged to create a visible positive emotional feedback loop. For instance, awarding honorary badges to users who complete specific training plans and converting their completion data into emotional incentives can foster a tighter connection between online-induced emotional experiences and offline behaviors.

The study identified that when daily OFC use exceeds 30 min, issues like information overload and emotional incentive fatigue can undermine the positive effects of OFC. Consequently, implementing a usage duration reminder system—such as monitoring user screen time and providing periodic alerts—can help mitigate information overload and cognitive fatigue caused by excessive use, promoting focused and efficient behavioral translation. Integrating “Healthy social networking sites Use” into public health education systems, including health curricula and public service announcements, is essential to enhance university students’ efficiency in using OFC health information and their awareness of behavioral translation.

## Conclusion

By extending the TPB framework, this study uncovers the “double-edged sword” effect of OFC in bridging the intention-behavior gap in resistance training among university students. Theoretically, this research breaks through the limitations of the traditional TPB, which overemphasizes cognitive drivers and individual proactive planning, by establishing the core roles of the “technology empowerment” and “emotional activation” dual pathways in the intention-behavior translation. OFC significantly promotes the transformation of intentions into behaviors through its influence on content, incentive facilitation for planning, and induction of positive emotions. However, when average daily usage exceeds 30 min, the promotive effect of OFC reverses, exhibiting a significant suppressing effect. Within the planning pathway, information overload impairs the efficiency of plan formulation and execution. Within the emotional pathway, the formation of a “reward cycle” causes the emotional experience to shift from being a “driver” to an “inhibitor,” leading users to become engrossed in the OFC content itself. This not only crowds out actual time for behavior but also undermines the foundational conditions necessary for behavioral translation.

## Data Availability

The original contributions presented in the study are included in the article/supplementary material, further inquiries can be directed to the corresponding author.

## References

[ref1] Abildso C. G. Daily S. M. Umstattd Meyer M. R. Perry C. K. Eyler A. (2023). Prevalence of meeting aerobic, muscle-strengthening, and combined physical activity guidelines during leisure time among adults, by rural-urban classification and region — United States, 2020. Am. J. Transplant. 23, 443–446. doi: 10.1016/j.ajt.2023.01.021, PMID: 36740195

[ref2] Ajzen I. (2005). Attitudes, personality and behaviour. (2nd ed.). London: McGraw-Hill Education.

[ref3] Ajzen I. (2011). The theory of planned behaviour: reactions and reflections. Psychol. Health 26, 1113–1127. doi: 10.1080/08870446.2011.613995, PMID: 21929476

[ref4] Ali I. Danaee M. Firdaus A. (2019). Social networking sites usage & needs scale (SNSUN): a new instrument for measuring social networking sites’ usage patterns and needs. J. Inf. Telecommun. 4, 151–174. doi: 10.1080/24751839.2019.1675461

[ref5] Armitage C. J. (2005). Can the theory of planned behavior predict the maintenance of physical activity? Health Psychol. 24, 235–245. doi: 10.1037/0278-6133.24.3.235, PMID: 15898858

[ref6] Bai S. Yin Y. Chen S. (2023). The impact of physical activity on electronic media use among Chinese adolescents and urban-rural differences. BMC Public Health 23:1264. doi: 10.1186/s12889-023-16103-x, PMID: 37386377 PMC10308688

[ref7] Bener A. Verjee M. Dafeeah E. E. Falah O. Al-Juhaishi T. Schlogl J. . (2013). Psychological factors: anxiety, depression, and somatization symptoms in low back pain patients. J. Pain Res. 6, 95–101. doi: 10.2147/jpr.S40740, PMID: 23403693 PMC3569050

[ref8] Bennie J. A. De Cocker K. Teychenne M. J. Brown W. J. Biddle S. J. H. (2019). The epidemiology of aerobic physical activity and muscle-strengthening activity guideline adherence among 383,928 U.S. adults. Int. J. Behav. Nutr. Phys. Act. 16:34. doi: 10.1186/s12966-019-0797-2, PMID: 30999896 PMC6472085

[ref9] Bennie J. A. De Cocker K. Tittlbach S. (2021). The epidemiology of muscle-strengthening and aerobic physical activity guideline adherence among 24,016 German adults. Scand. J. Med. Sci. Sports 31, 1096–1104. doi: 10.1111/sms.13922, PMID: 33464669

[ref10] Blozis S. A. Craft M. (2024). Alternative covariance structures in mixed-effects models: addressing intra- and inter-individual heterogeneity. Behav. Res. Methods 56, 2013–2032. doi: 10.3758/s13428-023-02133-1, PMID: 37231325 PMC11327215

[ref11] Boer M. Stevens G. W. Finkenauer C. van den Eijnden R. J. (2022). The complex association between social media use intensity and adolescent wellbeing: a longitudinal investigation of five factors that may affect the association. Comput. Hum. Behav. 128:107084. doi: 10.1016/j.chb.2021.107084

[ref12] Bosnjak M. Ajzen I. Schmidt P. (2020). The theory of planned behavior: selected recent advances and applications. Eur. J. Psychol. 16, 352–356. doi: 10.5964/ejop.v16i3.3107, PMID: 33680187 PMC7909498

[ref13] Bottaro R. Valenti G. D. Faraci P. (2024). Internet addiction and psychological distress: can social networking site addiction affect body uneasiness across gender? A mediation model. Eur. J. Psychol. 20, 41–62. doi: 10.5964/ejop.10273, PMID: 38487602 PMC10936664

[ref14] Brenner P. S. DeLamater J. D. (2014). Social desirability bias in self-reports of physical activity: is an exercise identity the culprit? Soc. Indic. Res. 117, 489–504. doi: 10.1007/s11205-013-0359-y

[ref15] Cameron D. S. Bertenshaw E. J. Sheeran P. (2015). The impact of positive affect on health cognitions and behaviours: a meta-analysis of the experimental evidence. Health Psychol. Rev. 9, 345–365. doi: 10.1080/17437199.2014.923164, PMID: 27028049

[ref16] Cao J. Liu F. Shang M. Zhou X. (2021). Toward street vending in post COVID-19 China: social networking services information overload and switching intention. Technol. Soc. 66:101669. doi: 10.1016/j.techsoc.2021.101669, PMID: 34898759 PMC8646579

[ref17] Cavaliere J. (2024). The perfect home workout plan. Available online at: https://www.athleanx.com/the-perfect-home-workout-plan (Accessed April 24, 2024).

[ref18] Cavarretta D. Hall E. Bixby W. (2018). The acute effects of resistance exercise on affect, anxiety, and mood – practical implications for designing resistance training programs. Int. Rev. Sport Exerc. Psychol. 12. doi: 10.1080/1750984X.2018.1474941

[ref19] Cavarretta D. J. Hall E. E. Bixby W. R. (2019). Affective responses from different modalities of resistance exercise: timing matters! Front. Sports Act. Living 1:5. doi: 10.3389/fspor.2019.00005, PMID: 33344929 PMC7739567

[ref20] Cheung G. W. Rensvold R. B. (2002). Evaluating goodness-of-fit indexes for testing measurement invariance. Struct. Equ. Modeling 9, 233–255. doi: 10.1207/S15328007SEM0902_5

[ref21] Churchill G. A. Jr. Peter J. P. (1984). Research design effects on the reliability of rating scales: a meta-analysis. J. Mark. Res. 21, 360–375. doi: 10.1177/002224378402100402

[ref22] Conner M. Norman P. (2022). Understanding the intention-behavior gap: the role of intention strength. Front. Psychol. 13:923464. doi: 10.3389/fpsyg.2022.923464, PMID: 35992469 PMC9386038

[ref23] Domin A. Ouzzahra Y. Vögele C. (2022). Features and components preferred by adolescents in smartphone apps for the promotion of physical activity: focus group study. JMIR Hum. Factors 9:e33972. doi: 10.2196/33972, PMID: 35679113 PMC9227785

[ref24] Durau J. Diehl S. Terlutter R. (2022). Motivate me to exercise with you: the effects of social media fitness influencers on users' intentions to engage in physical activity and the role of user gender. Digit. Health 8:20552076221102769. doi: 10.1177/20552076221102769, PMID: 35615268 PMC9125114

[ref25] Exelmans L. Van den Bulck J. (2017). Bedtime, shuteye time and electronic media: sleep displacement is a two-step process. J. Sleep Res. 26, 364–370. doi: 10.1111/jsr.12510 28271575

[ref26] Fornell C. Larcker D. F. (1981). Evaluating structural equation models with unobservable variables and measurement error. J. Mark. Res. 18, 39–50. doi: 10.2307/3151312

[ref27] Frison E. Eggermont S. (2020). Toward an integrated and differential approach to the relationships between loneliness, different types of Facebook use, and adolescents’ depressed mood. Commun. Res. 47, 701–728. doi: 10.1177/0093650215617506

[ref28] Ganson K. T. Nguyen L. Ali A. R. H. Hallward L. Jackson D. B. Testa A. . (2023). Associations between social media use, fitness- and weight-related online content, and use of legal appearance- and performance-enhancing drugs and substances. Eat. Behav. 49:101736. doi: 10.1016/j.eatbeh.2023.101736, PMID: 37141803

[ref29] Gao H. Tate M. Zhang H. Chen S. Liang B. (2018). Social media ties strategy in international branding: an application of resource-based theory. J. Int. Mark. 26, 45–69. doi: 10.1509/jim.17.0014

[ref30] Gao Y. Wang J. Liu C. (2021). Social media's effect on fitness behavior intention: perceived value as a mediator. Soc. Behav. Pers. 49, 1–11. doi: 10.2224/sbp.10300

[ref31] Garcia-Hermoso A. López-Gil J. F. Ramírez-Vélez R. Alonso-Martínez A. M. Izquierdo M. Ezzatvar Y. (2023). Adherence to aerobic and muscle-strengthening activities guidelines: a systematic review and meta-analysis of 3.3 million participants across 32 countries. Br. J. Sports Med. 57, 225–229. doi: 10.1136/bjsports-2022-106189, PMID: 36418149

[ref32] Geng L. Jiang G. Yu L. Xu Y. Huang W. Chen Z. . (2023). The most popular commercial weight management apps in the Chinese app store: analysis of quality, features, and behavior change techniques. JMIR Mhealth Uhealth 11:e50226. doi: 10.2196/50226, PMID: 37999950 PMC10709793

[ref33] Gholamnia-Shirvani Z. Ghofranipour F. Gharakhanlou R. Kazemnejad A. (2018). “Women and active life”: an extended TPB-based multimedia software to boost and sustain physical activity and fitness of Iranian women. Women Health 58, 834–850. doi: 10.1080/03630242.2017.1342739, PMID: 28682184

[ref34] Goldenberg A. Gross J. J. (2020). Digital emotion contagion. Trends Cogn. Sci. 24, 316–328. doi: 10.1016/j.tics.2020.01.009, PMID: 32160568

[ref35] Hair J. F. (1998). Multivariate data analysis. Upper Saddle River, NJ: Prentice Hall. Available at: https://books.google.com.hk/books?id=-ZGsQgAACAAJ

[ref36] Hall J. Kearney M. Xing C. (2018). Two tests of social displacement through social media use. Inf. Commun. Soc. 22, 1396–1413. doi: 10.1080/1369118X.2018.1430162

[ref37] Hall J. A. Liu D. (2022). Social media use, social displacement, and well-being. Curr. Opin. Psychol. 46:101339. doi: 10.1016/j.copsyc.2022.101339, PMID: 35395533

[ref38] Herman J. K. (2024). Influence of social media on physical activity engagement in Indonesia. Am. J. Recreat. Sports 3, 34–45. doi: 10.47672/ajrs.2047

[ref39] Jones M. V. Lane A. M. Bray S. R. Uphill M. Catlin J. (2005). Development and validation of the sport emotion questionnaire. J. Sport Exerc. Psychol. 27, 407–431. doi: 10.1123/jsep.27.4.407

[ref40] Kaplan A. M. Haenlein M. (2010). Users of the world, unite! The challenges and opportunities of social media. Bus. Horiz. 53, 59–68. doi: 10.1016/j.bushor.2009.09.003

[ref41] Kawashima M. Uchino M. Yokoi N. Uchino Y. Dogru M. Komuro A. . (2016). The association of sleep quality with dry eye disease: the Osaka study. Clin. Ophthalmol. 10, 1015–1021. doi: 10.2147/opth.S99620 27330271 PMC4898440

[ref42] Klier K. Rommerskirchen T. Brixius K. (2022). Fitspiration: a comparison of the sport-related social media usage and its impact on body image in young adults. BMC Psychol. 10:320. doi: 10.1186/s40359-022-01027-9, PMID: 36575554 PMC9793811

[ref43] Kong W. Song S. Zhao Y. C. Zhu Q. Sha L. (2021). TikTok as a health information source: assessment of the quality of information in diabetes-related videos. J. Med. Internet Res. 23:e30409. doi: 10.2196/30409, PMID: 34468327 PMC8444042

[ref44] Kontostoli E. Jones A. P. Pearson N. Foley L. Biddle S. J. H. Atkin A. J. (2023). The association of contemporary screen behaviours with physical activity, sedentary behaviour and sleep in adolescents: a cross-sectional analysis of the millennium cohort study. Int. J. Behav. Med. 30, 122–132. doi: 10.1007/s12529-022-10077-7, PMID: 35275347 PMC9879798

[ref45] Lachman M. E. Burack O. R. (2016). Planning and control processes across the life span: an overview. Int. J. Behav. Dev. 16, 131–143. doi: 10.1177/016502549301600203

[ref46] Lau P. W. C. Wang J. J. Ransdell L. L. Shi L. (2022). The effectiveness of Facebook as a social network intervention to increase physical activity in Chinese young adults. Front. Public Health 10:912327. doi: 10.3389/fpubh.2022.912327, PMID: 35937270 PMC9354571

[ref47] Lavoie H. A. McVay M. A. Pearl R. L. Fisher C. L. Jake-Schoffman D. E. (2025). The impact of fitness influencers on physical activity outcomes: a scoping review. J. Technol. Behav. Sci. doi: 10.1007/s41347-025-00513-2

[ref48] Lidong W. Xiuhong L. Kai Q. Donghai W. (2025). Examining the impact of perceived behavioral control and planning on closing the exercise intention-behavior gap: insights from a meta-analytic structural equation modeling study. Psychol. Sport Exerc. 78:102822. doi: 10.1016/j.psychsport.2025.102822, PMID: 39952422

[ref49] Little T. D. (1997). Mean and covariance structures (MACS) analyses of cross-cultural data: practical and theoretical issues. Multivar. Behav. Res. 32, 53–76. doi: 10.1207/s15327906mbr3201_3, PMID: 26751106

[ref50] Liu Z. Zhang Y. Zhang J. Song X. (2024). Run for the group: examining the effects of group-level social interaction features of fitness apps on exercise participation. Decis. Support. Syst. 187:114335. doi: 10.1016/j.dss.2024.114335

[ref51] López-Carril S. Bae D. Ribeiro T. Alguacil M. (2025). Social media as a driver of physical activity: a snapshot from sport sciences students. Perf. Enhanc. Health 13:100331. doi: 10.1016/j.peh.2025.100331

[ref52] Lutkenhaus R. O. Jansz J. Bouman M. P. (2019). Tailoring in the digital era: stimulating dialogues on health topics in collaboration with social media influencers. Digit. Health 5:2055207618821521. doi: 10.1177/2055207618821521, PMID: 30729023 PMC6350129

[ref53] MacCallum R. C. Browne M. W. Sugawara H. M. (1996). Power analysis and determination of sample size for covariance structure modeling. Psychol. Methods 1, 130–149. doi: 10.1037/1082-989X.1.2.130

[ref54] Maher C. Ryan J. Kernot J. Podsiadly J. Keenihan S. (2016). Social media and applications to health behavior. Curr. Opin. Psychol. 9, 50–55. doi: 10.1016/j.copsyc.2015.10.021

[ref55] Marino C. Gini G. Vieno A. Spada M. M. (2018). The associations between problematic Facebook use, psychological distress and well-being among adolescents and young adults: a systematic review and meta-analysis. J. Affect. Disord. 226, 274–281. doi: 10.1016/j.jad.2017.10.007, PMID: 29024900

[ref56] Martin C. (2023). 5 tips for building muscle. Available online at: https://www.cassandramartin.com/5-tips-for-building-muscle (Accessed April 24, 2024).

[ref57] Marty-Dugas J. Ralph B. C. W. Oakman J. M. Smilek D. (2018). The relation between smartphone use and everyday inattention. Psychol. Conscious. Theory Res. Pract. 5, 46–62. doi: 10.1037/cns0000131

[ref58] Mehri A. Solhi M. Garmaroudi G. Nadrian H. Sighaldeh S. S. (2016). Health promoting lifestyle and its determinants among university students in Sabzevar, Iran. Int. J. Prev. Med. 7:65. doi: 10.4103/2008-7802.180411 27141284 PMC4837801

[ref59] Middelweerd A. van der Laan D. M. van Stralen M. M. Mollee J. S. Stuij M. te Velde S. J. . (2015). What features do Dutch university students prefer in a smartphone application for promotion of physical activity? A qualitative approach. Int. J. Behav. Nutr. Phys. Act. 12:31. doi: 10.1186/s12966-015-0189-1, PMID: 25889577 PMC4359580

[ref60] Mohiyeddini C. Pauli R. Bauer S. (2009). The role of emotion in bridging the intention–behaviour gap: the case of sports participation. Psychol. Sport Exerc. 10, 226–234. doi: 10.1016/j.psychsport.2008.08.005

[ref61] O’Donnell N. Jerin S. I. Mu D. (2023). Using TikTok to educate, influence, or inspire? A content analysis of health-related EduTok videos. J. Health Commun. 28, 539–551. doi: 10.1080/10810730.2023.2234866, PMID: 37434532

[ref62] Oberiri Destiny A. Bahiyah O. Elif Asude T. Celestine Verlumun G. (2022). Information overload and misinformation sharing behaviour of social media users: testing the moderating role of cognitive ability. J. Inf. Sci. doi: 10.1177/01655515221121942

[ref63] Penglee N. Christiana R. W. Battista R. A. Rosenberg E. (2019). Smartphone use and physical activity among college students in health science-related majors in the United States and Thailand. Int. J. Environ. Res. Public Health 16:1315. doi: 10.3390/ijerph16081315, PMID: 31013703 PMC6517887

[ref64] Peters G. J. Y. (2014). The alpha and the omega of scale reliability and validity: why and how to abandon Cronbach's alpha and the route towards more comprehensive assessment of scale quality. Eur. Health Psychol.

[ref65] Picazo-Sánchez L. Domínguez-Martín R. García-Marín D. (2022). Health promotion on Instagram: descriptive–correlational study and predictive factors of influencers’ content. Int. J. Environ. Res. Public Health 19:15817. doi: 10.3390/ijerph192315817, PMID: 36497889 PMC9739539

[ref66] Pope Z. C. Barr-Anderson D. J. Lewis B. A. Pereira M. A. Gao Z. (2019). Use of wearable technology and social media to improve physical activity and dietary behaviors among college students: a 12-week randomized pilot study. Int. J. Environ. Res. Public Health 16:3579. doi: 10.3390/ijerph16193579, PMID: 31557812 PMC6801802

[ref67] Przybylski A. K. Weinstein N. (2017). A large-scale test of the goldilocks hypothesis. Psychol. Sci. 28, 204–215. doi: 10.1177/0956797616678438, PMID: 28085574

[ref68] Qin C. Li Y. Wang T. Zhao J. Tong L. Yang J. . (2024). Too much social media? Unveiling the effects of determinants in social media fatigue. Front. Psychol. 15:1277846. doi: 10.3389/fpsyg.2024.1277846, PMID: 39108425 PMC11300332

[ref69] Raggatt M. Wright C. J. C. Carrotte E. Jenkinson R. Mulgrew K. Prichard I. . (2018). “I aspire to look and feel healthy like the posts convey”: engagement with fitness inspiration on social media and perceptions of its influence on health and wellbeing. BMC Public Health 18:1002. doi: 10.1186/s12889-018-5930-7, PMID: 30097034 PMC6086030

[ref70] Ren Z. Zhang Y. Drenowatz C. Eather N. Hong J. Wang L. . (2024). How many adults have sufficient muscle-strengthening exercise and the associated factors: a systematic review consisting of 2,629,508 participants. J. Exerc. Sci. Fit. 22, 359–368. doi: 10.1016/j.jesf.2024.06.002, PMID: 39040428 PMC11261455

[ref71] Rhodes R. E. Dickau L. (2012). Experimental evidence for the intention–behavior relationship in the physical activity domain: a meta-analysis. Health Psychol. 31, 724–727. doi: 10.1037/a0027290, PMID: 22390739

[ref72] Rothman N. B. Magee J. C. (2016). Affective expressions in groups and inferences about members' relational well-being: the effects of socially engaging and disengaging emotions. Cogn. Emot. 30, 150–166. doi: 10.1080/02699931.2015.1020050, PMID: 25809798

[ref73] Ruangkanjanases A. Sivarak O. Wibowo A. Chen S. C. (2022). Creating behavioral engagement among higher education's prospective students through social media marketing activities: the role of brand equity as mediator. Front. Psychol. 13:1004573. doi: 10.3389/fpsyg.2022.1004573, PMID: 36304891 PMC9595281

[ref74] Scholz U. Schuz B. Ziegelmann J. P. Lippke S. Schwarzer R. (2008). Beyond behavioural intentions: planning mediates between intentions and physical activity. Br. J. Health Psychol. 13, 479–494. doi: 10.1348/135910707X216062, PMID: 17553212

[ref75] Searight H. R. Montone K. (2017). “Profile of mood states” in Encyclopedia of personality and individual differences. eds. Zeigler-Hill V. Shackelford T. K. (Springer International Publishing), 1–6. doi: 10.1007/978-3-319-28099-8_63-1

[ref76] Sheeran P. Webb T. L. (2016). The intention-behavior gap. Soc. Personal. Psychol. Compass 10, 503–518. doi: 10.1111/spc3.12265

[ref77] Sherman L. E. Hernandez L. M. Greenfield P. M. Dapretto M. (2018). What the brain 'likes': neural correlates of providing feedback on social media. Soc. Cogn. Affect. Neurosci. 13, 699–707. doi: 10.1093/scan/nsy051, PMID: 29982823 PMC6121147

[ref78] Snedden T. R. Scerpella J. Kliethermes S. A. Norman R. S. Blyholder L. Sanfilippo J. . (2019). Sport and physical activity level impacts health-related quality of life among collegiate students. Am. J. Health Promot. 33, 675–682. doi: 10.1177/0890117118817715 30586999 PMC7213817

[ref79] Sniehotta F. F. Scholz U. Schwarzer R. (2006). Action plans and coping plans for physical exercise: a longitudinal intervention study in cardiac rehabilitation. Br. J. Health Psychol. 11, 23–37. doi: 10.1348/135910705X43804, PMID: 16480553

[ref80] Sniehotta F. F. Schwarzer R. Scholz U. Schüz B. (2005). Action planning and coping planning for long-term lifestyle change: theory and assessment. Eur. J. Soc. Psychol. 35, 565–576. doi: 10.1002/ejsp.258

[ref81] Sokolova K. Perez C. (2021). You follow fitness influencers on YouTube. But do you actually exercise? How parasocial relationships, and watching fitness influencers, relate to intentions to exercise. J. Retail. Consum. Serv. 58:102276. doi: 10.1016/j.jretconser.2020.102276

[ref82] Sweet S. N. Brawley L. R. Hatchell A. Gainforth H. L. Latimer-Cheung A. E. (2014). Can persuasive messages encourage individuals to create action plans for physical activity? J. Sport Exerc. Psychol. 36, 413–423. doi: 10.1123/jsep.2013-0218, PMID: 25226610

[ref83] Twenge J. M. Martin G. N. (2020). Gender differences in associations between digital media use and psychological well-being: evidence from three large datasets. J. Adolesc. 79, 91–102. doi: 10.1016/j.adolescence.2019.12.018, PMID: 31926450

[ref84] Van der Linden S. (2017). The nature of viral altruism and how to make it stick. Nat. Hum. Behav. 1:0041. doi: 10.1038/s41562-016-0041

[ref85] Van Duin C. Sischka P. E. Heinz A. Willems H. (2025). The relationship between problematic social media use and health behavior: an exploratory specification curve analysis of large-scale survey data. Appl. Res. Qual. Life 20, 319–345. doi: 10.1007/s11482-024-10412-y

[ref86] Verduyn P. Ybarra O. Résibois M. Jonides J. Kross E. (2017). Do social network sites enhance or undermine subjective well-being? A critical review. Soc. Issues Policy Rev. 11, 274–302. doi: 10.1111/sipr.12033

[ref87] Wald A. Muennig P. A. O'Connell K. A. Garber C. E. (2014). Associations between healthy lifestyle behaviors and academic performance in U.S. undergraduates: a secondary analysis of the American College Health Association's National College Health Assessment II. Am. J. Health Promot. 28, 298–305. doi: 10.4278/ajhp.120518-QUAN-265 23941106

[ref88] Wang L. Feng W. Zhang J. Li T. (2024). Fitness or socializing - a multi-dimensional analysis of online fitness communities users. iScience 27:109753. doi: 10.1016/j.isci.2024.109753, PMID: 39040059 PMC11261065

[ref89] Wang Y. Wang Y. Greene B. Sun L. (2020). An analysis and evaluation of quality and behavioral change techniques among physical activity apps in China. Int. J. Med. Inform. 133:104029. doi: 10.1016/j.ijmedinf.2019.104029, PMID: 31759245

[ref90] Wei S. Chen X. Liu C. (2021). What motivates employees to use social media at work? A perspective of self-determination theory. Ind. Manag. Data Syst. 122, 55–77. doi: 10.1108/imds-06-2020-0322

[ref91] Wen Z. Ye B. (2014). Analyses of mediating effects: the development of methods and models. Adv. Psychol. Sci. 22:731. doi: 10.3724/SP.J.1042.2014.00731

[ref92] Westcott W. L. (2012). Resistance training is medicine: effects of strength training on health. Curr. Sports Med. Rep. 11, 209–216. doi: 10.1249/JSR.0b013e31825dabb8 22777332

[ref93] Weyland S. Finne E. Krell-Roesch J. Jekauc D. (2020). (How) does affect influence the formation of habits in exercise? Front. Psychol. 11:578108. doi: 10.3389/fpsyg.2020.578108, PMID: 33192892 PMC7645026

[ref94] Wiedmann K.-P. von Mettenheim W. (2020). Attractiveness, trustworthiness and expertise – social influencers’ winning formula? J. Prod. Brand. Manag. 30, 707–725. doi: 10.1108/JPBM-06-2019-2442

[ref95] Williams J. MacKinnon D. P. (2008). Resampling and distribution of the product methods for testing indirect effects in complex models. Struct. Equ. Model. 15, 23–51. doi: 10.1080/10705510701758166, PMID: 20179778 PMC2825896

[ref96] World Health Organization (2010) in Global recommendations on physical activity for health. ed. Whooley M. A.. 26180873

[ref97] World Health Organization. (2020). WHO guidelines on physical activity and sedentary behaviour. Available online at: https://www.who.int/publications/i/item/9789240015128 10.1136/bjsports-2020-102955 PMC7719906 33239350

[ref98] Yao Lin X. Lachman M. E. (2022). Associations between social media use, physical activity, and emotional well-being from the midlife in the United States refresher daily diary study. J. Aging Phys. Act. 30, 778–787. doi: 10.1123/japa.2021-0267, PMID: 34853182 PMC9156660

[ref99] Zhang W. H. Hsu M. C. Su R. H. (2020). Dances with structural equational modeling: a new generation episode. Xia men.

[ref100] Zhang X. Tang Q. Q. Cai Y. Y. (2024). What drives Chinese youth to use fitness-related health information on social media? An analysis of intrinsic needs, social media algorithms, and source credibility. Front. Public Health 12:1445778. doi: 10.3389/fpubh.2024.1445778, PMID: 39703487 PMC11655457

[ref101] Zhong L. Cao J. Xue F. (2024). The paradox of convenience: how information overload in mHealth apps leads to medical service overuse. Front. Public Health 12:1408998. doi: 10.3389/fpubh.2024.1408998, PMID: 39668954 PMC11634807

[ref102] Zielinski M. J. Veilleux J. C. Fradley M. F. Skinner K. D. (2023). Perceived emotion invalidation predicts daily affect and stressors. Anxiety Stress Coping 36, 214–228. doi: 10.1080/10615806.2022.2033973, PMID: 35135399 PMC9357853

